# Patient Disposition and Clinical Outcome After Referral to a Dedicated TAVI Clinic

**DOI:** 10.3389/fcvm.2019.00188

**Published:** 2020-01-10

**Authors:** Miroslawa Gorecka, Catriona Reddin, Gillian Madders, Laura Monaghan, Antoinette Neylon, Faisal Sharif, Brian Hynes, Evelyn Fennelly, Fiachra McHugh, Niamh Martin, Khalid Mohammed, Venu Reddy Bijjam, David Veerasingam, Alan Soo, Mark DaCosta, William Wijns, Darren Mylotte

**Affiliations:** ^1^Department of Cardiology, Galway University Hospital, SAOLTA Healthcare Group, Galway, Ireland; ^2^School of Medicine, National University of Ireland, Galway, Ireland; ^3^Department of Cardiothoracic Surgery, Galway University Hospital, SAOLTA Healthcare Group, Galway, Ireland; ^4^The Lambe Institute for Translational Medicine and Curam, SAOLTA University Healthcare Group, National University of Ireland Galway, Galway, Ireland

**Keywords:** aortic stenosis, transcatheter aortic valve implantation, TAVI, patient disposition, surgical aortic valve replacement, SAVR, optimal medical therapy, OMT

## Abstract

**Introduction:** Transcatheter aortic valve implantation (TAVI) is the standard of care for the majority of patients with severe symptomatic aortic stenosis (AS) at excessive-, high- and intermediate-surgical risk. A proportion of patients referred for TAVI do not undergo the procedure and proceed with an alternate treatment strategy. There is scarce data describing the final treatment allocation of such patients. Hence, we sought to evaluate the final treatment allocation of patients referred for TAVI in contemporary practice.

**Methods:** We performed a single center prospective observational study, including all patients referred to our institution for treatment of severe aortic stenosis between February 2014 and August 2017. Baseline demographic and clinical data were recorded. Patients were categorized according to treatment allocation: TAVI, surgical aortic valve replacement (SAVR) or optimal medical therapy (OMT). Clinical outcomes were adjudicated according to VARC-2 definitions. All patients were discussed at a dedicated Heart Team meeting.

**Results:** Total of 245 patients were referred for assessment to a dedicated TAVI clinic during the study period. Patients with moderate (*N* = 32; 13.1%) and asymptomatic (*N* = 31; 13.1%) AS were excluded. Subsequently, 53.9% (*N* = 132) received TAVI, 12.7% (*N* =31) were managed with OMT, and 7.3% (*N* =18) had SAVR. Reasons for OMT included primarily: patient's preference (*N* = 12; 38.7%); excessive surgical risk (*N* = 4; 12.9%) and severe frailty (*N* = 5; 16.1%). Reasons for surgical referral included low surgical risk (*N* = 11; 61.1%), excessive annulus size (*N* = 5; 27.8%), and aortic root dilatation (*N* = 2; 11.1%). Patients proceeding to SAVR had lower surgical risk than those in either the OMT or TAVI cohorts (*P* < 0.001). Mean STS score in SAVR group was 2.2 ± 1.3 vs. 4.5 ± 2.4 in OMT cohort and 6.1 ± 4.9 in TAVI cohort. Six-month all-cause mortality was 16.7, 19.4, and 9.3% among those receiving SAVR, OMT, and TAVI, respectively.

**Conclusions:** Almost half of all patients with severe AS referred to a dedicated TAVI clinic did not receive a TAVI. A considerable proportion of patients were reclassified as moderate AS (13%), were asymptomatic (13%), or intervention was determined to be futile (13%) due to advanced frailty. Early detection and increased awareness of valvular heart disease are required to increase the number of patients that can benefit from TAVI.

## Introduction

Aortic stenosis (AS) is the most common valve disease affecting elderly patients, occurring in ~3.4% of the population over 75 years of age (1). Transcatheter aortic valve implantation (TAVI) has transformed the management of AS patients and is considered to be the standard of care in elderly patients at excessive-, high- and intermediate-surgical risk. Indeed, TAVI has surpassed surgical aortic valve replacement (SAVR) as the dominant strategy for the treatment of symptomatic severe AS (2). National and regional differences in the availability of TAVI have emerged however and there exist considerable variations in the use of TAVI or SAVR (3).

Societal guidelines suggest that a Heart Team approach should facilitate the determination of the most appropriate therapeutic strategy for AS patients (4, 5). Such patients are usually referred to a dedicated Structural Heart clinic in a TAVI center for consideration of the most appropriate treatment. A proportion of patients with severe AS referred for TAVI are however likely to be more appropriately treated with SAVR due to young age and low operative risk, anatomical challenges, or concomitant coronary artery or mitral valve disease (4). Similarly, some patients at extreme-operative risk may be more appropriately treated conservatively. There is however, little information available on the final treatment allocation of patients referred to dedicated TAVI clinics (6, 7). Such information may have implications for healthcare resource allocation, service development planning, assessment of equitable patient access, and physician training.

We sought to address this knowledge gap by examining the disposition of patients referred to a dedicated TAVI clinic in contemporary clinical practice, to understand the motives for the chosen treatment allocation, and to describe clinical outcomes of various treatment strategies.

## Methods

### Patient Population

In this prospective single center study, data was collected on all patients with severe AS referred for assessment to a dedicated out-patient TAVI clinic between February 2014 and August 2017. Patients were referred from community medical practitioners, the general medical service, cardiologists, and cardiothoracic surgeons. The diagnosis of severe AS was reassessed in the clinic and patients with < severe AS were excluded from the study. Demographic, clinical, laboratory, echocardiographic, and procedural data were prospectively collected into a dedicated database. All patients provided informed consent for the procedure and the hospital ethical committee approved the data collection for this study.

Patients were categorized according to treatment allocation: TAVI, SAVR, or optimal medical therapy (OMT). The TAVI and SAVR groups included patients undergoing the respective intervention or those that died awaiting the procedure. The OMT group included patients treated with standard heart failure therapies, and balloon aortic valvuloplasty, but not deemed suitable for TAVI.

### Endpoints and Definitions

In all cases, treatment allocation and the rationale for this allocation was documented after Heart Team discussion. Echocardiographic data was defined according to established criteria (8). Severe AS was defined according to standard societal guidelines (mean pressure gradient >40 mmHg, aortic valve area by continuity equation < 1 cm^2^). In cases of suspected low-flow low-gradient AS, the diagnosis was confirmed using dobutamine stress echocardiography and/or multislice computed tomography. Severe pulmonary hypertension was defined as pulmonary artery systolic pressure ≥60 mmHg. Chronic kidney disease was defined as estimated glomerular filtration rate <30 mL/min/1.73 m^2^, chronic obstructive pulmonary disease as the ratio of forced expiratory volume in one second (FEV1) over forced vital capacity (FVC)–(FEV1/FVC) ≤70%. Obstructive coronary artery disease was defined as visual stenosis of a major epicardial artery ≥70% diameter stenosis.

Surgical risk was calculated using the Society of Thoracic Surgeons predicted risk of mortality (STS-PROM) score and the European System for Cardiac Operative Risk Evaluation (EuroSCORE; logistic EuroSCORE; and EuroSCORE II).

Clinical endpoints included procedural mortality, 30-day mortality, 6-month all-cause mortality, and stroke/transient ischemic attack as well as procedural complications. All outcomes were adjudicated according to the updated VARC-2 criteria (9). Clinical follow-up was performed by patient attendance at out-patient clinic or telephonic interview with patients, family members, and general practitioners. Follow-up time was the time between the procedure and follow-up in patients undergoing TAVI and SAVR or as the time from treatment decision in patients managed with OMT.

### Statistical Analysis

Continuous variables are presented as mean and standard deviation or median with interquartile range according to distribution. Normally distributed variables were compared with the Student *t*-test and non-normally distributed variables compared with the Wilcoxon rank-sum test. Categorical variables are presented as numbers and percentages, and were compared using chi-square or Fisher exact test. Multiple comparisons were analyzed using analysis of variance with Bonferroni correction or with the Kruskal-Wallis test. Survival was depicted using Kaplan-Meier graphs. Due to significant differences in baseline characteristics between treatment groups, we do not present comparative statistics on clinical outcomes. A probability value <0.05 was considered to indicate statistical significance. All analyses were performed with Minitab software version 17.

## Results

### Patients and Treatment Allocation

**Figure 1:**
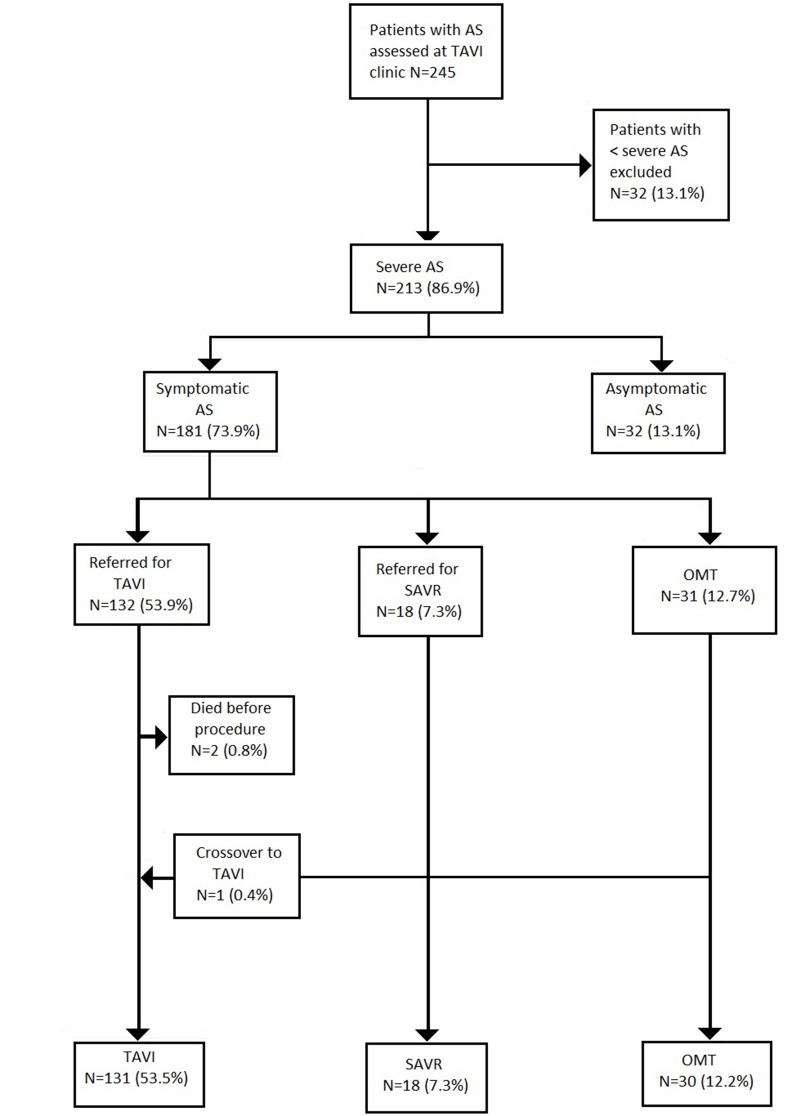
Patients flow. Values are number (%). AS, aortic stenosis; TAVI, transcatheter aortic valve implantation; SAVR, surgical aortic valve replacement; OMT, optimal medical therapy.

A total of 245 patients with AS were referred for assessment during the study period (Figure 1). Moderate AS was determined in 32 (13.1%) cases after careful multimodal imaging assessment. Among 213 patients with severe AS, 31.1% (*N* = 32) did not have symptoms, thus yielding a final study population of 181 patients with severe symptomatic AS. The median age of the study cohort was 83 [IQR 79–87] years and 53% (*N* = 96) were male (Table 1).

**Table 1:** Baseline characteristics according to treatment allocation.

**Demographic characteristics**	**All (*N =* 181)**	**TAVI (*N* = 132)**	**SAVR (*N* = 18)**	**OMT (*N* = 31)**	***p*-value**
Age, median [IQR]	83 [79–87]	83.3 [80.5–87]	73 [60–79]	86 [82–88]	<0.001^*^^#^^$^
Male sex	96 (53)	67 (50.8)	12 (66.7)	17 (54.8)	0.4
**Symptoms**					
NYHA Class III/IV	137 (77.8)	107 (84.3)	12 (66.7)	19 (61.3)	0.01^*^
Angina	42 (24.4)	35 (28)	4 (22.2)	3 (9.7)	0.1
Syncope	50 (28.4)	42 (33.1)	2 (11.1)	6 (19.4)	0.1
**Co-morbid conditions**					
Diabetes mellitus	40 (22.3)	29 (22.3)	5 (27.8)	6 (19.3)	0.8
Hypertension	147 (81.2)	116 (87.9)	10 (55.6)	21 (67.7)	<0.001^*^^#^
CKD eGFR <30 mL/min/1.73 m^2^	24 (13.9)	20 (15.6)	1(5.9)	3 (11.1)	0.4
COPD	26 (14.5)	23 (17.7)	1 (5.6)	2 (6.5)	0.1
PVD	29 (16.2)	28 (21.5)	0 (0)	1 (3.2)	0.001^*^^#^
Stroke	34 (19)	27 (20.8)	2 (11.1)	5 (16.1)	0.5
Prior MI	49 (27.7)	37 (28.7)	4 (22.2)	8 (26.7)	0.8
Prior PCI	55 (30.9)	42 (32.6)	4 (22.2)	9 (29)	0.6
Prior CABG	25 (14)	21 (16.2)	1 (5.6)	3 (10)	0.3
Atrial fibrillation	72 (39.8)	50 (38.2)	9 (50)	13 (41.9)	0.6
PASP > 60 mmHg	8 (4.5)	5 (3.9)	0 (0)	3 (9.7)	–
Permanent pacemaker	21 (11.8)	15 (11.5)	0 (0)	6 (20)	0.1
**Biological assessment**					
Weight, Kg	74.9 ± 16.4	74.7 ± 16	83.4 ± 17.2	70.3 ± 16.7	0.1
eGFR, mL/min/1.73 m^2^	51 [39–64.5]	50 [38.3–64]	77 [43–86.5]	51 [38–59]	0.02^#^^$^
**Pre TAVI Coronary angiography,**					
Obstructive CAD (>70% visual diameter stenosis)	57 (33.7)	41 (32.5)	5 (27.8)	11 (44)	0.8
PCI	20 (13.8)	23 (22.1)	0 (0)	7 (30.4)	0.01^#^^$^
**Echocardiography**					
Peak gradient, mmHg	75 [65–90]	75 [66–90]	70 [59–78.5]	72 [60–101]	N/A
Mean gradient, mmHg	47.5 [39–57]	48 [40–57.5]	41 [36–49.1]	48 [37.3–63.5]	N/A
AVA, cm^2^	0.7 [0.5–0.8]	0.6 [0.5–0.8]	0.7 [0.5–1.1]	0.7 [0.6–0.8]	N/A
LVEF, %	55 [50–60]	55 [50–60]	60 [55–60]	55 [40–60]	N/A
PASP, mmHg	35 [28–45]	34 [28–41]	37 [25.8–47]	40 [33.5–49.5]	N/A
MR Grade ≥ 3	32 (18.1)	29 (22.5)	2 (11.1)	1 (3.3)	N/A
**Surgical risk**					
EuroSCORE II	8.1 ± 8.5	9.2 ± 9.2	2.6 ± 1.6	6.4 ± 6.1	0.004^#^^$^
Logistic EuroSCORE	20.3 ± 15.3	22.9 ± 15.9	6.2 ± 3.7	17.8 ± 11.5	<0.001^#^^$^
STS PROM score	5.4 ± 4.6	6.1 ± 4.9	2.2 ± 1.3	4.5 ± 2.4	0.001^*^^#^^$^

*Values are number (%), median [interquartile range], or mean ± SD. p < 0.05 was used as the level of statistical significance*.

* *Denotes statistical significance in TAVI-OMT pairwise comparison*.

# *Denotes statistical significance in TAVI-SAVR pairwise comparison*.

$ *Denotes statistical significance in SAVR-OMT pairwise comparison. COPD, chronic obstructive pulmonary disease; PVD, peripheral vascular disease; MI, myocardial infarction; PCI, percutaneous coronary intervention; CABG, coronary artery bypass graft; PASP, pulmonary artery systolic pressure; CKD, chronic kidney disease; eGFR, estimated glomerular filtration rate; CAD, coronary artery disease; AVA, aortic valve area; MR, mitral regurgitation; LVEF, left ventricular ejection fraction; STS, Society of Thoracic Surgeons Predicted Risk of Mortality score*.

Treatment allocation after Heart Team discussion was as follows (Table 1): TAVI in 132 (53.9%); SAVR in 18 (9.9%); and OMT in 31 (17.1%). One patient initially managed with OMT proceeded to TAVI as a novel large THV (Medtronic, 34 mm Evolut R) became commercially available. Two patients died awaiting TAVI. Surgery was preferred to TAVI in 18 patients (7.3%) due to low surgical risk (*N* = 11; 61.1%)–mean STS score: 2.2%; excessive annulus size for commercially available TAVI devices at the time (*N* = 5; 27.8%); and bicuspid aortic valve morphology with aortic root dilatation (*N* = 2; 11.1%).

OMT was preferred in 31 cases (12.7%) due to a patient preference to avoid intervention (*N* = 12; 38.7%); excessive annulus size but too frail for surgery (*N* = 4; 12.9%); severe frailty or immobility (*N* = 5; 16.1%); end-stage pulmonary disease (*N* = 3; 9.7%); severe cognitive impairment (*N* = 2; 6.5%); and end-stage malignancy (*N* = 1; 3.2%). In 4 cases (12.9%), patients died before completion of their out-patient TAVI work-up. One patient (3.2%) did not attend follow-up (Table 2).

**Table 2:** Indication for treatment allocation to surgery or medical therapy.

**Treatment allocation**	***N* (%)**
Primary reason for OMT	***N*** **=** **31**
Patient preference	12 (38.7)
Excessive annulus size but too frail for surgery	4 (12.9)
Severe cognitive impairment	2 (6.5)
End-stage malignancy	1 (3.2)
End-stage pulmonary disease	3 (9.7)
Severe frailty / immobility	5 (16.1)
Did not attend clinic	1 (3.2)
Died before work-up complete	4 (12.9)
Primary reason for SAVR	***N*** **=** **18**
Low surgical risk	11 (61.1)
Excessive annulus size	5 (27.8)
Aortic root dilatation	2 (11.1)

*Values are number (%). OMT, optimal medical therapy; SAVR, surgical aortic valve replacement*.

### Demographic Information

Baseline demographic, clinical, biological and echocardiographic characteristics according to treatment allocation are presented in Table 1. Patients managed with SAVR were significantly younger than those in either the OMT or TAVI cohorts (73 [IQR 60–79] vs. 86 [IQR 82–88] and 83.3 [IQR 80.5–87], respectively; *P* < 0.001). As expected, SAVR patients had lower STS scores then the other groups (2.2 ± 1.3% vs. 4.5 ± 2.4% and 6.1 ± 4.9% respectively; *P* = 0.001). Patients undergoing SAVR also had higher median left ventricular ejection fraction than those in the OMT or the TAVI group [60% [IQR 55–60] vs. 55% [IQR 40–60] and 55% (50–60), respectively]. The median waiting time for TAVI was 70 [23–160] days, whereas median waiting time for SAVR was 272 [181–361] days (*P* < 0.001).

### Clinical Outcome

Clinical outcomes are presented in Table 3. SAVR was associated with numerically higher rates of bleeding (27.8% vs. 10.6%) and acute kidney injury (33.3% vs. 4.5%), than TAVI, but with lower rates of new permanent pacemaker insertion (11.1% vs. 28.9%) and vascular complications (0% vs. 9.1%).

**Table 3:** Procedural and clinical outcomes.

**Outcomes, *N* (%)**	**All** ** (*N* = 181)**	**TAVI** ** (*N* = 132)**	**SAVR** ** (*N* = 18)**	**OMT** ** (*N* = 31)**
Procedural mortality (*N* = 150)	2 (1.3)	1 (0.8)	1 (5.6)	N/A
30-day mortality	6 (3.3)	3 (2.3)	2 (11.1)	1 (3.2)
6 month all-cause morality	21 (11.8)	12 (9.3)	3 (16.7)	6 (19.4)
Stroke/TIA	6 (3.6)	3 (2.5)	1 (5.6)	2 (7.1)
**Procedural complications**				
Myocardial Infarction	1 (0.7)	0 (0)	1 (5.6)	N/A
Any bleeding	19 (12.7)	14 (10.6)	5 (27.8)	N/A
Life-threatening bleeding	8 (5.3)	5 (3.8)	3 (16.7)	N/A
Acute kidney injury	12 (8)	6 (4.5)	6 (33.3)	N/A
Any vascular complication	12 (8)	12 (9.1)	0 (0)	N/A
Major vascular complication	6 (4)	6 (4.5)	0 (0)	N/A
New permanent pacemaker	27 (18)	25 (18.9)	2 (11.1)	N/A

*Values are number (%). TAVI, transcatheter aortic valve implantation; SAVR, surgical aortic valve replacement; OMT, optimal medical therapy; TIA, transient ischaemic attack*.

**Figure 2:**
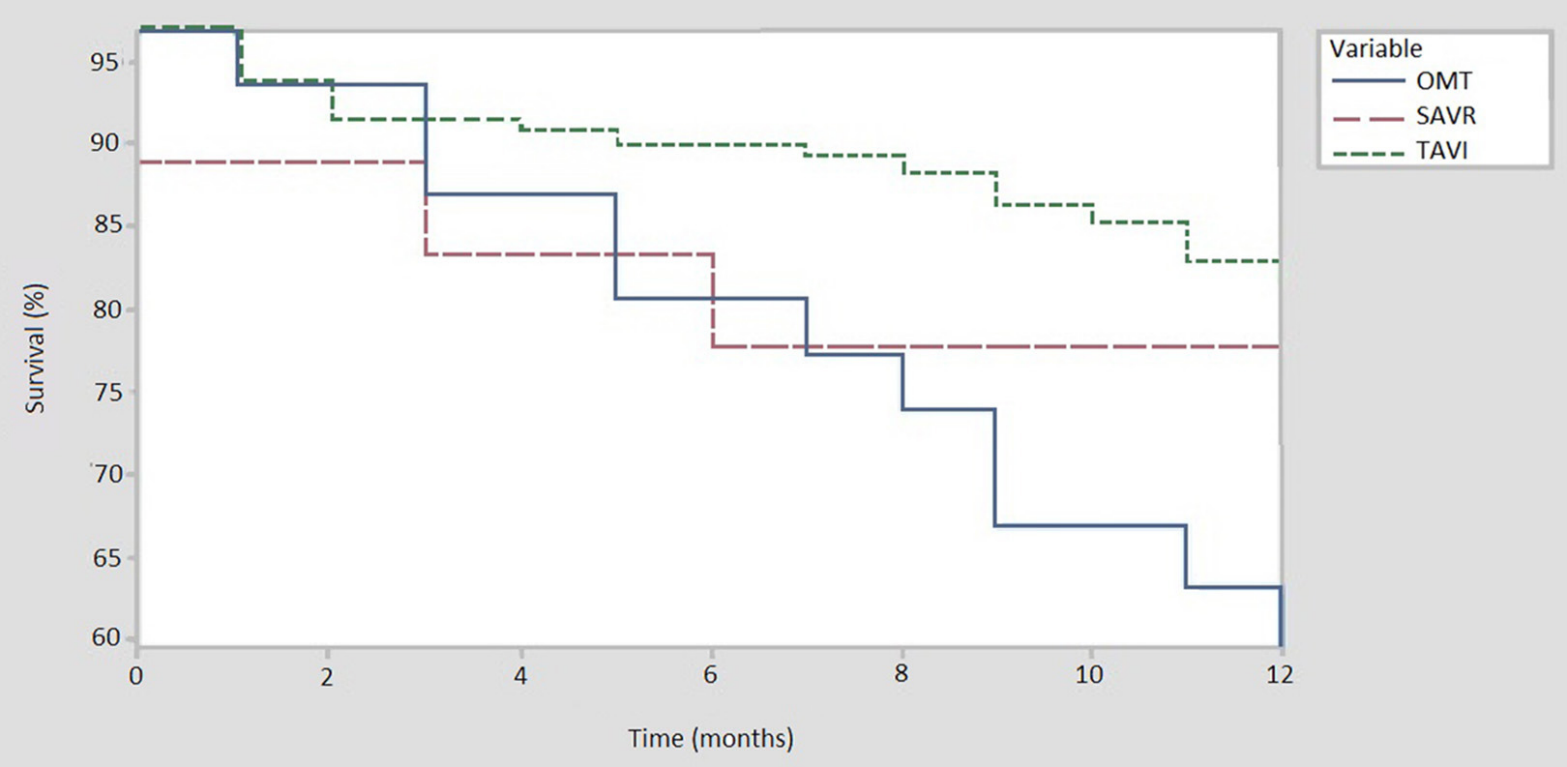
All cause mortality at 12 months. TAVI, trancatheter aortic valve implantation (*N* = 131); OMT, optimal medical therapy (*N* = 30); SAVR, sugical aortic valve replacement (*N* = 18).

When compared to TAVI, SAVR was associated with a numerically higher procedural (5.6% vs. 0.8%) and 30-day mortality (11.1% vs. 2.3%). Six-month follow-up data was available for all patients in the OMT and SAVR group, and for 129 (97.7%) patients in the TAVI cohort. Median follow-up was 14 months [IQR 7–22] in the OMT cohort, 16.5 months [IQR 7.5–27] in the SAVR group and 12 months [IQR 8–21.8] for TAVI patients. Six-month all-cause mortality was highest among patients managed with OMT as compared to SAVR or TAVI (19.4% vs. 16.7% vs. 9.3%, respectively). All-cause mortality is displayed using Kaplan-Meier survival curves in Figure 2.

## Discussion

The main findings of our study are that only 53.5% of patients referred to a dedicated clinic for consideration for TAVI went on to receive a transcatheter heart valve. After reassessment of the severity of AS, 13.1% were reclassified as moderate rather than severe AS and among severe AS patients, up to 13.1% were asymptomatic and did not proceed to intervention. Among symptomatic severe AS patients, 20% were unsuitable for TAVI, with 7.3% undergoing SAVR and a further 12.7% of cases were deemed futile and hence managed with OMT. This information has important implications.

It seems striking that in contemporary clinical practice, only one in two patients referred for TAVI actually receive this life-saving therapy. These results are however not unique, and other studies have documented similarly low rates of application of TAVI technology: 59% in an Italian study (*N* = 98) and 39% in a Canadian report (6, 7). These results must be contextualized however: when patients with moderate or asymptomatic AS were excluded, then nearly three-quarters (73%) were treated with TAVI. Further 10% were referred for SAVR.

In our study, more than 1 in 10 (13.1%) patients purportedly with severe AS were reclassified as moderate AS after assessment at a dedicated clinic. These data suggest that societal guidelines and position papers which recommend centralization of complex procedures, such as TAVI, at dedicated tertiary referral centers are appropriate (4, 10, 11). Centralization serves, not only to improve procedural outcome, but also more appropriately select the most appropriate intervention (if any) for a given patient. In AS, ancillary diagnostic capabilities such as transoesophageal and dobutamine stress echocardiography, and multislice computed tomography are required. Such techniques may not be readily available in smaller referring centers and could result in patients being misclassified, as demonstrated in our study.

There remain few data describing the prevalence of symptoms in elderly patients with severe AS referred for TAVI (6, 7). In our patient population, quality of life and functional capacity are often more important patient-related outcome measures than mortality (12, 13). Indeed, elderly patients are often reluctant to undertake procedures that confer a mortality advantage if symptomatic benefit is not guaranteed. In the current study, 13.1% of our elderly patients (mean age 84.7 years) with severe AS did not report cardiovascular symptoms. In selected cases, exercise stress testing was performed to confirm the absence of symptoms, but in many cases, additional testing was not performed as the patients were satisfied with their quality of life. Such treatment decisions are appropriate in this elderly population but are less relevant in younger AS patients where the mortality advantage of TAVI is more pertinent.

In patients treated with OMT (*N* = 31), 12 (38.7%) refused the procedure, 4 (12.9%) died before the decision was finalized and 4 (12.9%) did not proceed because of unsuitable anatomy (large annuli) and concomitant frailty. In all other cases, the procedure was deemed futile due to co-morbidities such as end-stage malignancy, excessive frailty, and cognitive impairment. These results are similar to previous studies in which the majority of patients who did not proceed to TAVI either declined the procedure, had unsuitable anatomy for percutaneous approach, or due to significant co-morbidities a symptomatic improvement was viewed as unlikely (6, 7). Patients managed with OMT in our cohort were older than patients in the two other subgroups, but interestingly, the prevalence of co-morbidities was not higher than in patients treated with TAVI or SAVR. The STS score of patients managed with OMT was significantly lower, than the mean STS score in the TAVI cohort (4.5 ± 2.4 vs. 6.1 ± 4.9 respectively; *P* = 0.04). As expected, patients referred for SAVR had the lowest STS score (2.2 ± 1.3; *P* < 0.001). The lower STS score in the OMT group compared to those undergoing TAVI may be attributed to factors that are not accounted for in the traditional surgical risk scores, such as frailty, cognitive impairment, etc. These treatment decisions highlight the important role of a multidisciplinary team (Heart Team) in determining management strategies. Moreover, these data point to the vital importance of considering patient's preference in decision making; nearly 4 in 10 patients treated with OMT refused TAVI. As expected, the prognosis in the OMT group was dismal with all-cause mortality of 19.4% at 6 months.

Despite TAVI now being extended to younger and lower risk populations, almost 1 in 10 patients (*N* = 18; 9.9%) in the current cohort were referred for SAVR. The recent publication of two low risk TAVI randomized trials will change these practices and will result in many younger patients being referred for TAVI in the coming years (14, 15). In our study, surgery was preferential in many cases, irrespective of operative risk, due to the presence of bicuspid aortic valve and dilation of the aortic root or concomitant severe coronary artery disease. Surgery will remain an important treatment option for patients with infective endocarditis, aortic thrombus or other anatomic characteristics that render TAVI unsuitable, or those with coexisting multivalve disease amenable to surgical correction (4). In contrast, the 28% of severe AS patients referred for SAVR due to excessively large aortic annuli not suitable for commercially available TAVI systems at that time would be expected to have TAVI in contemporary practice since larger devices such as the Medtronic Evolut R 34 mm (Medtronic, Minneapolis, Minnesota, USA) or overexpansion of the SAPIEN 3 (Edwards Lifesciences, Irvine, CA, USA) prosthesis have emerged.

In a significant proportion of patients, frailty and cognitive impairment may limit the symptomatic benefit derived from TAVI (16, 17). Intervention in such patients is deemed to be “futile.” It is important to acknowledge however, that in many such cases, a delayed presentation or diagnosis may have contributed to patients being labeled as futile. Opportunistic screening for severe valvular heart disease by general practitioners in the community has the potential to reduce the number of patients presenting late and improve outcome (18). A heart valve disease awareness survey performed among patients above the age of 60 years in nine European countries, found that only 7% of patients could identify symptoms of AS correctly and in 54.2% of cases, their general practitioner did not routinely use a stethoscope to examine their heart (19). It is recommended that all patients age ≥70 years should undergo opportunistic cardiovascular examination for a systolic murmur, symptoms of AS, and a referral for a transthoracic echocardiography if a murmur is detected (20). Community events, such as European Heart Valve Disease Awareness day serve to raise awareness of AS among general population and encourage seeking medical advice at an earlier stage (21).

## Limitations

The current study comprises a single center experience of a small number of patients. Furthermore, changes in the threshold for intervention have evolved during the study enrolment which would have affected the disposition of patients at intermediate risk. Indeed, recent data suggesting extension of TAVI to patients at low operative risk will further impact patient disposition in the future. Consideration will need to be given to valve durability and the risk of paravalvular leak especially in this younger, lower risk cohort. Advancements in TAVI technology and patient screening, and local awareness of the dedicated TAVI clinic are also likely to have impacted the proportion of patients assigned to TAVI or OMT.

Our main objective was to report the ultimate treatment allocation for this patient population. Nevertheless, we also provide clinical outcome data according to the VARC definitions, but we did not present statistical comparisons between treatment groups due to considerable differences in the baseline characteristics of these patient populations. Interpretation of outcome data should be interpreted with caution, since the sample size is small and selection bias was introduced in the screening process.

## Conclusions

Almost half of all patients with severe AS referred to a dedicated clinic for TAVI do not receive a transcatheter heart valve. A considerable proportion of these elderly patients are reclassified as moderate AS, are asymptomatic, or intervention is determined to be futile due to advanced frailty or cognitive impairment. Early detection and increased awareness of valvular heart disease are required to reduce the proportion of patients declined TAVI.

## Data Availability Statement

All datasets generated for this study are included in the article/supplementary material.

## Ethics Statement

Ethical approval for this study has been granted by Ethics Committee, Galway University Hospital, Galway, Ireland.

## Author Contributions

MG was responsible for data collection, statistical analysis, literature review, and write up of the manuscript. CR, GM, and LM were responsible for data collection. AN, FS, BH, DV, AS, and MD were responsible for patient management. EF, FM, NM, KM, and VB were responsible for literature review and review of the manuscript. WW was responsible for review of the manuscript. DM was responsible for patient management, review of the manuscript, and approval of the final version of the manuscript.

### Conflict of Interest

MD is a Proctor and Consultant for Medtronic and Microport. The remaining authors declare that the research was conducted in the absence of any commercial or financial relationships that could be construed as a potential conflict of interest.
